# Bilateral transcutaneous auricular vagus nerve stimulation for misophonia symptoms: a case report and review of the literature

**DOI:** 10.3389/fpsyg.2026.1903444

**Published:** 2026-07-15

**Authors:** Francesca Proietti, Massimo Marano, Emanuele Rizzo, Jae-Jun Song, Vincenzo Di Lazzaro, Giuliano Albergo

**Affiliations:** 1 Unit of Neurology, Neurophysiology, Neurobiology and Psychiatry, Department of Medicine, University Campus Bio-Medico of Rome, Rome, Italy; 2 Fondazione Policlinico Universitario Campus Bio-Medico, Rome, Italy; 3 Department of Otorhinolaryngology-Head and Neck Surgery, Korea University Medical Center, Seoul, Republic of Korea

**Keywords:** autonomic dysregulation, autonomic hyperarousal, case report, misophonia, neuromodulation, psychological distress, vagus nerve stimulation

## Abstract

Misophonia is characterized by a markedly decreased tolerance to specific sounds, often accompanied by intense emotional, behavioral and autonomic responses. Current treatment evidence remains limited, and neuromodulation-based approaches targeting autonomic dysregulation are largely unexplored. This case report describes a 64-year-old woman with severe misophonic symptoms, whose main trigger was the rhythmic thudding sound of a basketball being bounced nearby. Exposure to this sound elicited intense distress, palpitations, choking sensation, dyspnea, acute anxiety, terror and an overwhelming urge to escape, leading to avoidance and functional impairment. The patient was receiving stable sertraline treatment and underwent a 1-month protocol of bilateral transcutaneous auricular vagus nerve stimulation, delivered daily for 60 min at an individually adjusted, non-painful intensity. Misophonia severity, psychological distress, anxiety, depressive symptoms, sleep, circadian rhythm and mood-related symptoms were assessed at baseline, after treatment and at 1-month follow-up. The Duke Misophonia Questionnaire Symptoms Composite Scale decreased from 80 at baseline to 9 after treatment and 5 at follow-up, indicating sustained reduction in symptom severity. Overall psychological distress, somatization, anxiety, hostility and depressive symptoms also improved. Sleep quality improved after treatment but returned to baseline at follow-up. Conversely, functional impairment decreased immediately after treatment but increased at follow-up, suggesting a dissociation between symptom reduction and perceived functional recovery. This case suggests that bilateral transcutaneous auricular vagus nerve stimulation may be a feasible adjunctive intervention for misophonia characterized by prominent autonomic hyperarousal. Findings are preliminary and hypothesis-generating and controlled studies are needed to clarify its therapeutic role.

## Introduction

Misophonia is a chronic condition characterized by a markedly decreased tolerance to specific sounds or associated stimuli, which trigger intense emotional responses, such as anxiety, anger or disgust, together with autonomic responses (Ferrer-Torres and Giménez-Llort, 2022). According to the Expert Consensus Definition (Swedo et al., 2022), misophonic triggers commonly include human-generated repetitive sounds, such as chewing, eating, swallowing, pen clicking, keyboard typing or footsteps. Reactions to these stimuli are typically disproportionate to the physical characteristics of the sound and may lead to significant distress and impairment in social, occupational and personal functioning (Kumar et al., 2017; Schröder et al., 2013; Swedo et al., 2022).

Although misophonia is increasingly recognized as a clinically relevant condition, its nosological status remains debated, and it is not formally included in the Diagnostic and Statistical Manual of Mental Disorders (DSM-5-TR) (Schröder et al., 2013; Swedo et al., 2022). This diagnostic uncertainty reflects, at least in part, the heterogeneous clinical presentation of misophonia, which may include affective, cognitive, behavioral, sensory and physiological components (Swedo et al., 2022; Neacsiu et al., 2022). Patients may experience not only immediate emotional reactions to trigger sounds, but also anticipatory anxiety, hypervigilance, avoidance behaviors, interpersonal difficulties and functional limitations (Schröder et al., 2013; Swedo et al., 2022; Quek et al., 2018; Wu et al., 2014; Zhou et al., 2017). These features suggest that misophonia cannot be fully understood as a simple auditory intolerance, but rather as a complex condition involving interactions between sound processing, emotional salience, autonomic regulation and behavioral responses (Kumar et al., 2017; Neacsiu et al., 2022).

Several neurobiological models have attempted to explain these mechanisms. The neurophysiological model proposed by Jastreboff and Jastreboff (2023) suggests that misophonia may be sustained by abnormally strong functional connections between the auditory system and limbic-autonomic networks. Consistently, neuroimaging studies have identified heightened activity in the anterior insular cortex and altered salience network connectivity in response to misophonic triggers (Kumar et al., 2017; Neacsiu et al., 2022). The anterior insula and anterior cingulate are involved in interoceptive awareness, emotional salience and autonomic regulation and may therefore contribute to the intense visceral and physiological responses frequently reported by patients with misophonia, including increased heart rate, muscle tension, and heightened arousal (Kumar et al., 2017; Swedo et al., 2022).

The autonomic dimension of misophonia is particularly relevant. Trigger sounds can elicit marked physiological activation, and patients often describe bodily reactions such as palpitations, dyspnea, muscular tension, agitation and an urgent need to escape. Grossini et al. (2022) suggested that misophonia may involve dysregulated interaction between auditory limbic and autonomic regulatory pathways, with excessive sympathetic activation and reduced parasympathetic modulation. Given the role of the vagus nerve in parasympathetic regulation, these findings indirectly support the hypothesis that impaired vagal regulation may contribute to misophonia-related emotional and autonomic dysregulation.

On this basis, neuromodulatory approaches targeting vagal pathways have recently gained increasing attention. Transcutaneous auricular vagus nerve stimulation (taVNS) is a non-invasive technique that stimulates the auricular branch of the vagus nerve and has been proposed as a method for modulating central networks and influencing brain regions involved in emotional regulation, interoception and salience processing (Farmer et al., 2021; Zhang et al., 2024; Tyler, 2025). By potentially enhancing parasympathetic regulation and reducing autonomic hyperarousal, taVNS may represent a promising adjunctive strategy for conditions characterized by emotional and physiological dysregulation. However, its potential role in misophonia remains largely unexplored.

Current treatment evidence for misophonia remains limited and heterogeneous. To date, there is no established evidence-based pharmacological or neuromodulation-based treatment for misophonia. Available interventions mainly include cognitive-behavioral, exposure-based, sound-focused, third-wave behavioral and pharmacological approaches targeting associated symptoms, but the level of evidence varies substantially across modalities (Ferrer-Torres and Giménez-Llort, 2022; Mattson et al., 2023). Among these, cognitive-behavioral therapy currently has the strongest preliminary support, including evidence from a randomized clinical trial, whereas neuromodulation-based approaches remain largely unexplored (Jager et al., 2020; Mattson et al., 2023). This limited treatment landscape supports the exploration of mechanism-informed adjunctive interventions, particularly in patients with severe symptoms and prominent autonomic hyperarousal. On this basis, this article reports a patient with severe misophonic symptoms treated with bilateral taVNS as an adjunctive intervention and discusses its potential role within a mechanism-informed treatment model.

## Case description

A 64-year-old woman with 16 years of education underwent a comprehensive clinical evaluation for misophonic symptoms. At assessment, she was receiving stable pharmacological treatment with sertraline 50 mg once daily, with no medication changes during the observation period.

During the clinical interview, the patient reported a marked intolerance to auditory stimuli characterized by a deep, muffled and repetitive quality. Her main trigger was the rhythmic thudding sound of a basketball being bounced nearby. Exposure to this stimulus elicited intense distress and prominent autonomic symptoms, including palpitations, choking sensation, dyspnea, acute anxiety, terror and overwhelming urge to escape. These symptoms caused significant interference with daily functioning and led to avoidance of situations in which the trigger sound could occur. No clinically relevant physical examination findings were reported. Clinically relevant findings were derived from the clinical interview and psychometric assessment and included severe sound-triggered autonomic arousal, avoidance behavior and functional impairment.

### Timeline

The timeline of clinical assessment, intervention and follow-up is shown in Table 1.

**Table 1:** Timeline of clinical assessment, intervention and follow-up.

Time point	Clinical history and symptoms	Treatment/assessment
1996–2004	The patient reported long-standing sensitivity to noise, although this had not previously been associated with the same level of distress and functional impairment observed during the current episode.	Previous long-term psychotherapy was reported in adulthood, with no specific improvement in misophonia symptoms.
2024	The patient attended a specialized tinnitus and misophonia center because of increasing sound intolerance.	No structured misophonia-specific intervention was reported.
2025	Symptoms markedly worsened after repeated exposure to a deep, muffled, rhythmic thudding sound produced by a basketball being bounced nearby. The sound became particularly disturbing when perceived in the bedroom area. Exposure elicited intense autonomic and emotional reactions, including palpitations, choking sensation, dyspnea, acute anxiety, terror, anger, helplessness and urge to escape. Anticipatory anxiety persisted even after the sound stopped, driven by fear that the trigger would recur.	Sertraline treatment was initiated and titrated to 50 mg/day.
December 2025—baseline clinical assessment	The patient underwent clinical evaluation for severe misophonic symptoms. During the assessment, exposure to similar thudding sounds was reported to be associated with marked distress and autonomic arousal.	Baseline psychometric assessment was performed using the DMQ, SCL-90-R, BDI-II, STAI-X1, STAI-X2, SCRAM-r.
December 2025—day 1–30	The patient underwent home-based bilateral taVNS.	Daily 60-min bilateral taVNS sessions were delivered, with stimulation applied to internal surface of the tragus and cymba conchae, at 30 Hz, 1000 μs, with individually adjusted non-painful intensity.
January 2026—T0	The patient reported reduced physiological reactivity to trigger-like sounds and appeared more relaxed during clinical reassessment.	Post-treatment psychometric assessment was performed.
February 2026—T1	Symptom severity remained reduced, although perceived functional impairment increased again, possibly related to anticipatory anxiety, hypervigilance and fear of symptom recurrence after discontinuation of stimulation.	Follow-up psychometric assessment was performed. Sertraline remained unchanged.

BDI-II, Beck Depression Inventory-II; DMQ, Duke Misophonia Questionnaire; SCL-90-R, Symptom Checklist-90-Revised; SCRAM-r, revised Sleep, Circadian Rhythms and Mood questionnaire; STAI-X1, State Anxiety Inventory; STAI-X2, Trait Anxiety Inventory; taVNS, transcutaneous auricular vagus nerve stimulation; T0, post-treatment assessment; T1, 1-month follow-up.

### Diagnostic assessment

The clinical diagnosis of misophonia was based on clinical interview, clinical phenomenology and DMQ assessment. The clinical interview focused on the nature of the trigger sounds, the emotional and autonomic reactions elicited by the exposure, avoidance behaviors, functional impairment and temporal relationship between trigger exposure and symptom onset. Conditions potentially overlapping with sound intolerance or anxiety-related symptoms, including tinnitus, hyperacusis and panic-like anxiety responses, were clinically considered. Other relevant psychiatric symptoms, including depressive and anxiety symptoms were assessed through clinical interview and standardized self-report measures. Nevertheless, the symptom pattern was judged to be primarily consistent with misophonia because reactions were selectively elicited by a specific repetitive trigger sound and were associated with anger, autonomic arousal, avoidance and functional impairment. No structured psychiatric diagnostic interview was administered. No diagnostic access-related challenges were reported. No specific prognostic characteristics were applicable to this single-case report.

The patient was assessed at three time points: baseline, after 1 month of bilateral taVNS treatment (T0), and 1 month after discontinuation of stimulation (T1). Misophonia severity and functional impairment were assessed using the Duke Misophonia Questionnaire (DMQ) (Rosenthal et al., 2021), which was considered the primary outcome measure. The DMQ is a self-report questionnaire assessing misophonia-related symptoms and impairment over the previous 30 days. The instrument includes an initial trigger checklist assessing sensitivity to different auditory and visual stimuli. Subsequent sections assess the frequency of misophonic reactions and symptom domains including emotional responses, physiological responses, cognitive responses, coping and avoidance behaviors before, during and after exposure to trigger sounds, functional impairment across social, occupational, relational and daily-life domains, and misophonia-related beliefs. Also, it comprises two subscales, the Symptoms Composite Scale and the Impairment Scale.

General psychological distress was evaluated using the Symptom Checklist-90-R (SCL-90-R) (Derogatis and Savitz, 1999), a multidimensional self-report questionnaire assessing a broad range of psychological symptoms. It consisted of several subscales, such as somatization, depression, anxiety, hostility and a Global Severity Index that provides an overall measure of current psychological distress. Depressive symptoms were assessed with the Beck Depression Inventory-II (BDI-II) (Beck et al., 1996), a self-report measure of depressive symptom severity. State and trait anxiety were assessed using the State-Trait Anxiety Inventory (STAI-X1 and STAI-X2) (Spielberger et al., 1983), which separately evaluates current anxiety state and dispositional anxiety proneness. Sleep quality, circadian rhythm characteristics and mood-related symptoms were assessed using the revised Sleep, Circadian Rhythms and Mood questionnaire (SCRAM-r) (Di Pompeo et al., 2024), considering sleep quality, chronotype, depression and anxiety dimensions.

### Therapeutic intervention

Given the persistence of severe misophonic symptoms and functional impairment despite ongoing selective serotonin reuptake inhibitors (SSRI) pharmacotherapy, together with the absence of established evidence-based pharmacological or neuromodulation-based therapies for misophonia, bilateral taVNS was offered on an individual compassionate basis as an adjunctive intervention. This intervention was selected because of the patient's prominent autonomic symptomatology and hypothesized involvement of autonomic dysregulation in misophonia (Kumar et al., 2017; Schröder et al., 2017; Grossini et al., 2022).

Following an initial training session on correct device placement, application and operation, the patient underwent a 1-month bilateral taVNS protocol, using the Healon Pro device (Neurive, Seoul, Korea). Stimulation was delivered bilaterally through conductive ear tips positioned at the internal surface of the tragus and cymba conchae. The protocol consisted of daily 60-min sessions for 30 consecutive days, performed at home. The patient received painless bilateral taVNS at an individually adjusted intensity, based on the sensory threshold and eliciting a non-painful tingling sensation. Stimulation frequency was set at 30 Hz, with a pulse width of 1000 μs. No changes were made to ongoing sertraline treatment during the stimulation protocol or follow-up. Adherence and tolerability were assessed by patient self-report during post-treatment and follow-up clinical assessments. The patient reported completing the home-based stimulation protocol, and no adverse effects were reported.

### Follow-up and outcomes

The 1-month bilateral taVNS protocol, administered alongside stable sertraline treatment, was associated with a marked reduction in misophonia symptoms and overall psychological distress. The DMQ Symptoms Composite Scale decreased from 80 at baseline to 9 at T0, corresponding to an 88.7% reduction, and further decreased to 5 at T1. The DMQ Impairment Scale decreased from 13 to 4 at T0, but increased to 28 at T1, suggesting a rebound in perceived functional impairment despite sustained symptom improvement.

General psychological distress also improved. The SCL-90-R Global Severity Index decreased from 67.6 to 45.4 at T0 and remained stable at T1. Somatization, anxiety, hostility and depression scores showed similar reductions, remaining below baseline at follow-up. Additional measures showed decreased depressive symptoms and state-trait anxiety. SCRAM-r scores indicated improved sleep quality during treatment, followed by a return to baseline at T1, whereas mood-related SCRAM-r subscales showed sustained improvement.

Results are shown in Table 2.

**Table 2:** Psychometric assessment at baseline, after 1 month of bilateral taVNS treatment and at 1-month follow-up.

Measure	Baseline	T0	T1
DMQ symptoms composite scale	80	9	5
DMQ impairment	13	4	28
SCL-90-R somatization	73.12	43.41	49.37
SCL-90-R depression	69.79	52.31	48.81
SCL-90-R anxiety	79.73	41.89	41.89
SCL-90-R hostility	80	46.67	42.5
SCL-90-R global severity index	67.6	45.4	46.45
SCRAM-r quality of sleep	9	15	9
SCRAM-r chronotype	14	9	12
SCRAM-r depression	13	9	7
SCRAM-r anxiety	16	4	4
BDI-II	8	3	4
STAI-X1	28	23	23
STAI-X2	48	31	34

BDI-II, Beck Depression Inventory-II; DMQ, Duke Misophonia Questionnaire; SCL-90-R, Symptom Checklist-90-Revised; SCRAM-r, revised Sleep, Circadian Rhythms and Mood questionnaire; STAI-X1, State Anxiety Inventory; STAI-X2, Trait Anxiety Inventory; taVNS, transcutaneous auricular vagus nerve stimulation; T0, post-treatment assessment; T1, 1-month follow-up.

## Discussion

**Figure 1:**
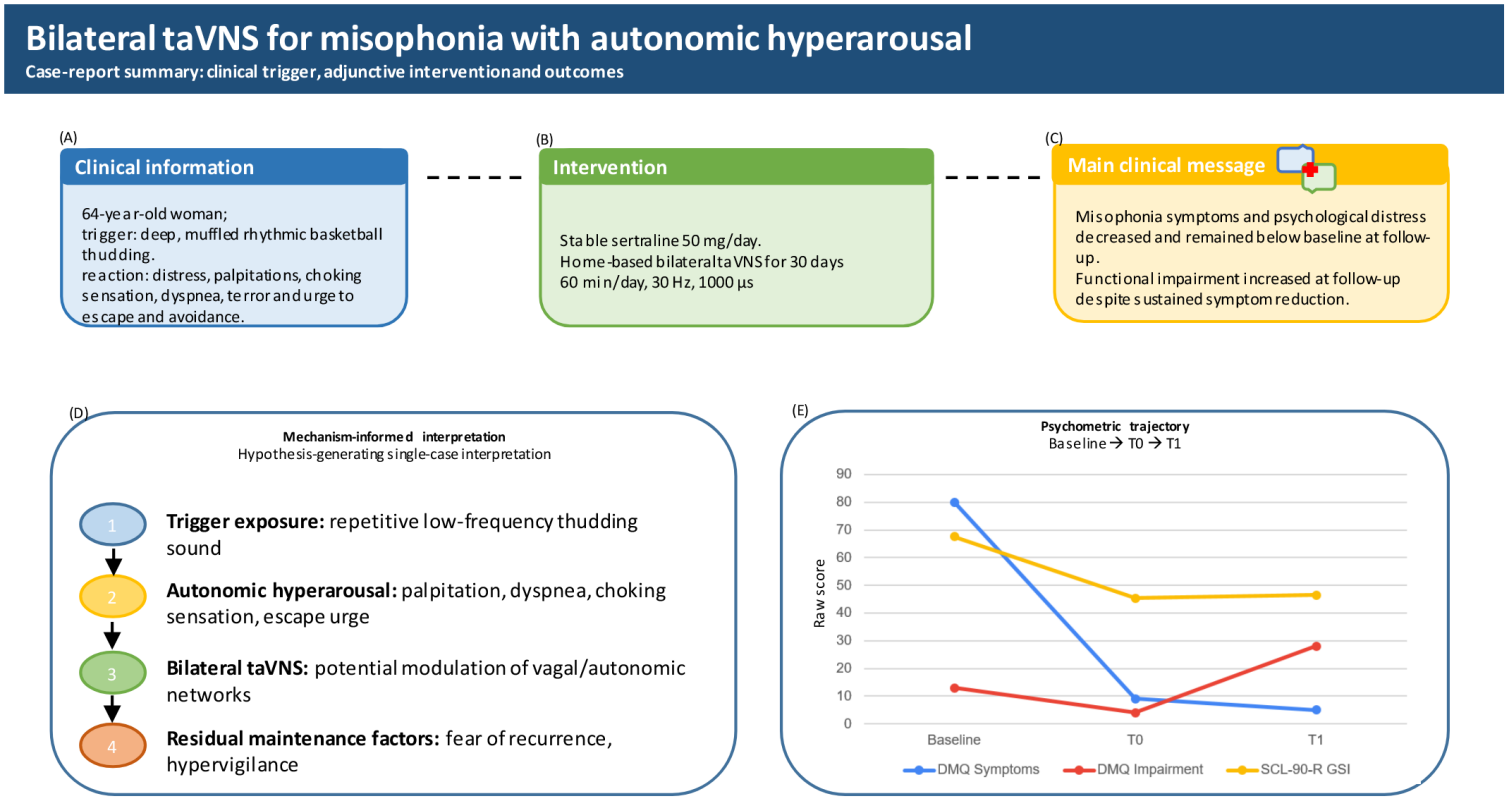
Case-summary figure of bilateral transcutaneous auricular vagus nerve stimulation (taVNS) for misophonia symptoms with autonomic hyperarousal. **(A)** Clinical information. **(B)** Adjunctive intervention. **(C)** Main clinical message. **(D)** Hypothesis-generating mechanism-informed interpretation. **(E)** Psychometric trajectory across baseline, post-treatment (T0) and 1-month follow-up (T1). Raw scores are shown for the Duke Misophonia Questionnaire (DMQ) Symptoms Composite, DMQ Impairment Scale, and Symptom Checklist-90-Revised Global Severity Index (SCL-90-R GSI).

This case report describes a patient with severe misophonic symptoms characterized by prominent autonomic hyperarousal, in whom a 1-month protocol of bilateral taVNS, administered alongside stable sertraline treatment, was associated with a marked reduction in misophonia symptom severity. The improvement was maintained 1 month after discontinuation of stimulation. Reductions were also observed across several domains of psychological distress, including anxiety, hostility, somatization, depressive symptoms and global distress. Sleep quality improved during the stimulation period, although this effect was not maintained at follow-up. In contrast, functional impairment followed a different trajectory, decreasing immediately after treatment but increasing again at follow-up (Figure 1). This pattern suggests that symptom severity and perceived functional recovery may not necessarily improve in parallel.

A possible interpretation is that taVNS may have contributed to a reduction in autonomic hyperreactivity to misophonic triggers. This interpretation is clinically plausible because the patient's misophonic episodes were characterized by marked autonomic symptoms. The reduction in somatization and anxiety scores may therefore reflect a decrease in neurovegetative symptoms associated with autonomic hyperactivation. Similarly, improvements in hostility and sleep quality during the stimulation period may suggest a broader stabilization of emotional and physiological regulation.

This interpretation is consistent with current neurobiological models of misophonia, which implicate abnormal interactions between auditory processing systems, salience-related networks, limbic emotional circuits and autonomic regulatory pathways (Kumar et al., 2017; Neacsiu et al., 2022). Grossini et al. (2022) further suggested that misophonia may involve altered orthosympathetic-parasympathetic balance, supporting the hypothesis that autonomic dysregulation may represent a clinically relevant mechanism in at least a subgroup of patients. Given the role of the vagus nerve in parasympathetic regulation, taVNS may help modulate central autonomic networks and attenuate exaggerated emotional and physiological responses to misophonic triggers (Breit et al., 2018; Yakunina et al., 2017; Farmer et al., 2021; Zhang et al., 2024). However, this mechanistic interpretation remains speculative in the present case, as no objective physiological measures were collected. Therefore, it cannot be determined whether the clinical improvement observed after stimulation was accompanied by measurable changes in autonomic functioning.

The increase in functional impairment observed at follow-up, despite sustained improvement in misophonia symptom severity, is particularly important because it complicates a simple interpretation of treatment response. This pattern suggests a possible dissociation between trigger-related physiological reactivity and behavioral functioning. Although the patient reported a substantial reduction in physiological reactivity to trigger sounds, she also described renewed fear of symptom recurrence after discontinuing stimulation. This fear appeared to be associated with anticipatory anxiety, hypervigilance and avoidance behavior, leading to greater perceived limitation in daily activities. These findings suggest that reducing autonomic hyperreactivity may not be sufficient to restore functioning when cognitive-behavioral maintenance mechanisms, such as fear of recurrence, avoidance and maladaptive appraisals, persist.

This dissociation should be interpreted in the context of the limited and heterogeneous treatment literature on misophonia. A recent systematic review identified only 33 studies specifically addressing misophonia treatment, including one randomized controlled trial, one open-label trial and 31 case reports or case series, highlighting the early stage and limited methodological rigor of the field (Mattson et al., 2023). Current approaches include cognitive-behavioral interventions, exposure-based and sound-focused strategies, third-generation psychological therapies and pharmacological treatments targeting comorbid symptoms or associated emotional dysregulation, although the level of evidence varies substantially across modalities (Ferrer-Torres and Giménez-Llort, 2022; Mattson et al., 2023). To date, no established evidence-based pharmacological or neuromodulation-based treatment exists for misophonia. Among available interventions, CBT currently has the strongest preliminary support. CBT-based approaches are clinically relevant because misophonia is maintained not only by auditory triggers, but also by anticipatory anxiety, hypervigilance, avoidance, maladaptive appraisals and difficulty regulating trigger-related emotional and psychological responses. The strongest evidence comes from the randomized controlled trial by Jager et al. (2020), in which group-based CBT reduced misophonia severity compared with a waitlist condition, with effects maintained at follow-up. Additional support comes from open-label and case-based studies, including interventions incorporating psychoeducation, cognitive restructuring, reduction of avoidance and strategies to manage physiological arousal (Schröder et al., 2017; Bernstein et al., 2013; Mattson et al., 2023; Dover and McGuire, 2023; Roushani and Honarmand, 2021; Zarotti et al., 2022). Further case-based and pilot evidence has described CBT-based, exposure-related, EMDR, pharmacological, and broader clinical approaches to misophonia, although the evidence remains preliminary (Altinöz et al., 2018; Alekri and Al Saif, 2019; Brout et al., 2018; Jager et al., 2021; Cecilione et al., 2022).

From this perspective, taVNS should not be considered an alternative to psychological treatment, but rather a potential adjunctive intervention within a mechanism-informed treatment model. taVNS may be particularly relevant for patients whose misophonia is characterized by prominent autonomic hyperarousal, whereas CBT-based strategies may be needed to address hypervigilance, fear of recurrence, avoidance behaviors, safety behaviors and functional impairment. In the present case, this integrated model is supported by the observed dissociation between sustained symptom reduction and rebound in perceived impairment after stimulation discontinuation.

Other treatment approaches may also be relevant depending on the patient's dominant maintaining mechanisms. Exposure-based and counter-conditioning strategies may help reduce conditioned responses to specific triggers, but they should be tailored carefully because misophonia often involves anger, disgust, irritation and autonomic arousal rather than fear alone (Swedo et al., 2022; Kumar et al., 2017; Ferrer-Torres and Giménez-Llort, 2022; Dozier, 2015a; Dozier, 2015b; Dozier, 2015c). Sound-focused approaches, including tinnitus retraining therapy, sound therapy, white noise generators and masking strategies, may reduce attention to or reactivity toward trigger sounds, particularly in patients with comorbid tinnitus or hyperacusis, although controlled evidence remains limited and these interventions may not fully address the cognitive, emotional behavioral and autonomic complexity of misophonia (Jastreboff and Jastreboff, 2023; Mattson et al., 2023). Third-generation psychological therapies, including mindfulness-based interventions, acceptance and commitment therapy and dialectical behavior therapy components, may help patients modify their relationship with anger, disgust, anxiety, shame and urge to escape or avoid, but current evidence remains preliminary and mainly based on case reports or small studies (Kamody and Del Conte, 2017; Schneider and Arch, 2017; Palumbo et al., 2018; Hayes, 2004; Hayes et al., 2005). Pharmacological evidence is similarly limited and largely based on case reports. SSRIs, including fluoxetine and sertraline, have been described in individual cases, usually targeting associated anxiety, depressive or obsessive-compulsive symptoms rather than misophonia-specific mechanisms (Sarigedik and Yurteri, 2021; Vidal et al., 2017; Zuschlag and Leventhal, 2021). Other reported strategies include methylphenidate, low-dose risperidone and propranolol in selected clinical contexts (Osuagwu et al., 2020; Naguy et al., 2022; Webb, 2022; Mattson et al., 2023). In the present case, the persistence of severe symptoms and functional impairment despite ongoing SSRI treatment further supported the exploration of taVNS on an individual compassionate basis.

Overall, this case suggests that bilateral taVNS may be a feasible adjunctive intervention for misophonia characterized by prominent autonomic hyperarousal. However, given the single-case design, absence of a sham control condition, concomitant sertraline treatment, short follow-up, lack of adherence monitoring and absence of objective autonomic measures, the findings remain preliminary and hypothesis-generating. In addition, the interpretation of both symptom improvement and the rebound in functional impairment remains speculative, as no objective autonomic measures or behavioral indices of avoidance were collected. Because the autonomic dysregulation represented the core rationale for taVNS in this case, the absence of physiological measures, such as heart rate variability, electrodermal activity, or heart rate, represents a major limitation. The concurrent use of sertraline represents an additional confounding factor. Although the dose remained stable throughout the stimulation protocol and follow-up, its contribution to symptom improvement cannot be excluded, particularly with respect to anxiety, distress and emotional reactivity. Therefore, the observed changes should be interpreted as occurring during adjunctive taVNS rather than as being attributable to taVNS alone. Future sham-controlled studies with large samples, longer follow-up, standardized outcome measures, adherence monitoring and physiological indices such as heart rate variability and electrodermal activity are needed to clarify whether taVNS has a specific therapeutic role in misophonia, which patients may be most likely to benefit, and whether combining taVNS with CBT-based interventions may improve both symptom severity and functional recovery.

### Patient perspective

The patient reported a subjective reduction in the intensity of physiological reactions to trigger sounds during the stimulation period, with perceived improvement in emotional control and daily tolerability. After discontinuation of stimulation, however, she described increased fear of symptom recurrence and renewed avoidance, despite sustained reduction in symptom severity.

## Data Availability

The original contributions presented in the study are included in the article/supplementary material, further inquiries can be directed to the corresponding author/s.
