# Supplementary material for: Bilateral transcutaneous auricular vagus nerve stimulation for misophonia symptoms: a case report and review of the literature
Source: Front Psychol. 2026 Jul 15;17:1903444. doi: 10.3389/fpsyg.2026.1903444 (PMC13414741; doi:10.3389/fpsyg.2026.1903444)
Supplement: Supplementary file 1 [file Table_1.docx]

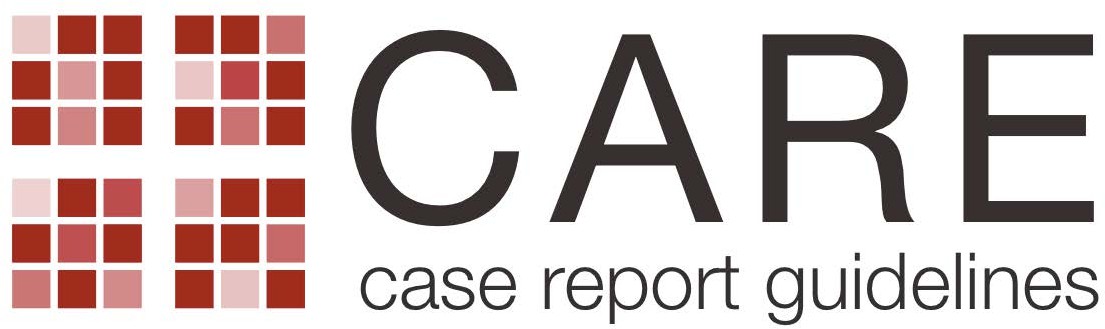
CARE Checklist of information to include when writing a case report
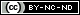


**Topic Item Checklist item description Reported in section**

**Title 1** The diagnosis or intervention of primary focus followed by the words “case report” . . . . . . . . . . . . . . . . . . Title page

**Key Words 2** 2 to 5 key words that identify diagnoses or interventions in this case report, including "case report" Title page

**Abstract**

**(no references)**

**3a** Introduction: What is unique about this case and what does it add to the scientific literature? Abstract

**3b** Main symptoms and/or important clinical findings . . . . . . . . . . . . . . . . . . . . . . . . . . . . . . . . . . . . . . . . . . . . . . . . . . . Abstract

**3c** The main diagnoses, therapeutic interventions, and outcomes Abstract

**3d** Conclusion—What is the main “take-away” lesson(s) from this case? Abstract

**Introduction 4** One or two paragraphs summarizing why this case is unique (**may include** reference**s**) Introduction

**Patient Information 5a** De-identified patient specific information Case description

**5b** Primary concerns and symptoms of the patient Case description

**5c** Medical, family, and psycho-social history including relevant genetic information Introduction

**5d** Relevant past interventions with outcomes Introduction; table 1

**Clinical Findings**

**Timeline**

**Diagnostic Assessment**

**Therapeutic Intervention**

**Follow-up and Outcomes**

1. Describe significant physical examination (PE) and important clinical findings Case description
2. Historical and current information from this episode of care organized as a timeline Table 1

**8a** Diagnostic testing (such as PE, laboratory testing, imaging, surveys). Diagnostic assessment

**8b** Diagnostic challenges (such as access to testing, financial, or cultural) Diagnostic assessment

**8c** Diagnosis (including other diagnoses considered) Diagnostic assessment

**8d** Prognosis (such as staging in oncology) where applicable Diagnostic assessment

**9a** Types of therapeutic intervention (such as pharmacologic, surgical, preventive, self-care) . . . . . . . . . . . . . . . . . Therapeutic intervention

**9b** Administration of therapeutic intervention (such as dosage, strength, duration) Therapeutic intervention

**9c** Changes in therapeutic intervention (with rationale) Therapeutic intervention

**10a** Clinician and patient-assessed outcomes (if available) Follow-up and outcomes; table 2

**10b** Important follow-up diagnostic and other test results Follow-up and outcomes; table 2

**10c** Intervention adherence and tolerability (How was this assessed?) Follow-up and outcomes

**10d** Adverse and unanticipated events Follow-up and outcomes

**Discussion 11a** A scientific discussion of the strengths AND limitations associated with this case report Discussion

**11b** Discussion of the relevant medical literature **with references** Discussion

**11c** The scientific rationale for any conclusions (including assessment of possible causes) Discussion

**11d** The primary “take-away” lessons of this case report (without references) in a one paragraph conclusion Discussion

**Patient Perspective 12** The patient should share their perspective in one to two paragraphs on the treatment(s) they received . . . . Patient perspective

**Informed Consent 13** Did the patient give informed consent? Please provide if requested . . . . . . . . . . . . . . . . . . . . . . . . . . . . . . . . . . . . . . **Yes X** **No
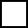
**
